# Co-expression Network Approach Reveals Functional Similarities among Diseases Affecting Human Skeletal Muscle

**DOI:** 10.3389/fphys.2017.00980

**Published:** 2017-12-01

**Authors:** Kavitha Mukund, Shankar Subramaniam

**Affiliations:** ^1^Department of Bioengineering, University of California, San Diego, La Jolla, CA, United States; ^2^Departments Cellular and Molecular Medicine, Computer Science and Engineering, University of California, San Diego, La Jolla, CA, United States

**Keywords:** co-expression networks, functional module framework, bioenergetics, calcium signaling, skeletal muscle physiology, neuromuscular disease, drug repurposing, human protein interaction network

## Abstract

Diseases affecting skeletal muscle exhibit considerable heterogeneity in intensity, etiology, phenotypic manifestation and gene expression. Systems biology approaches using network theory, allows for a holistic understanding of functional similarities amongst diseases. Here we propose a co-expression based, network theoretic approach to extract functional similarities from 20 heterogeneous diseases comprising of dystrophinopathies, inflammatory myopathies, neuromuscular, and muscle metabolic diseases. Utilizing this framework we identified seven closely associated disease clusters with 20 disease pairs exhibiting significant correlation (*p* < 0.05). Mapping the diseases onto a human protein-protein interaction network enabled the inference of a common program of regulation underlying more than half the muscle diseases considered here and referred to as the “protein signature.” Enrichment analysis of 17 protein modules identified as part of this signature revealed a statistically non-random dysregulation of muscle bioenergetic pathways and calcium homeostasis. Further, analysis of mechanistic similarities of less explored significant disease associations [such as between amyotrophic lateral sclerosis (ALS) and cerebral palsy (CP)] using a proposed “functional module” framework revealed adaptation of the calcium signaling machinery. Integrating drug-gene information into the quantitative framework highlighted the presence of therapeutic opportunities through drug repurposing for diseases affecting the skeletal muscle.

## Introduction

Human skeletal muscle is a versatile tissue, with structure and function governed by complex interactions between its sensing, signaling, force transduction, metabolic and basic cellular processing machinery (transcription and translation) (Kierszenbaum and Tres, 2015). Precisely coordinated activity between each of its components is essential for muscle health and normal functioning of associated motor activity. A disruption to any component within this complex system of interactions leads to disorders of the muscle, typically characterized by muscle fiber loss, reduced motor output and possibly death. Epidemiological, clinical, and physiological studies have contributed immensely to our understanding of pathogenesis and manifestation of individual muscle diseases, revealing similarities amongst them (Jones and Round, 1990; Askanas and Engel, 2008).

Recent advancements in genomic technologies have enabled newer opportunities for understanding mechanisms that are common and distinct across muscle pathologies. High-throughput measurements, multiscale phenotypic data and integrative analysis are beginning to provide increasingly comprehensive understanding of muscle dynamics, specifically for biomarker discovery (Laaksonen et al., 2006; Dewey et al., 2011; Azuaje et al., 2012; Gupta et al., 2014). However, very few studies have developed and implemented techniques for extracting similarities underlying muscle diseases, on a much broader scale. For instance, Blandin et al. (2013) utilized the yeast-two hybrid (Y2H) methodology combined with co-expression networks to generate a muscular LGMD-centered interaction network (LGMD-Limb girdle muscular dystrophy) identifying a total of 1,018 proteins connected by 1,492 direct binary interactions enriched for cytoskeletal and extracellular matrix protein interactions.

In this study, we propose a quantitative framework to assess relationships between 20 diseases affecting the muscle which, based on their pathological/clinical presentation, were categorized into dystrophinopathies, inflammatory myopathies, neuromuscular and metabolic diseases of the muscle (Table 1, see Methods) (Engel and Franzini-Armstrong, 2004). Briefly, dystrophinopathies include Emery-Dreifuss muscular dystrophies (EDMD), limb girdle muscular dystrophies (LGMDs), Duchenne muscular dystrophy (DMD) and Becker muscular dystrophy (BMD). While EDMDs and LGMDs are caused by mutations in muscle structural genes, DMD and BMD are caused by frame shift and in-frame mutations respectively, of the DMD gene. Progressive weakening and wasting of skeletal muscle characterize all these diseases. The inflammatory myopathies considered include polymyositis (PM), dermatomyositis (DM), juvenile dermatomyositis (JDM), inclusion body myositis (IBM) and hereditary inclusion body myositis (HIBM). These myopathies are caused primarily by infiltration of immune cells and are characterized by chronic inflammation and weakening of muscle. Metabolic diseases of the muscle including the mitochondrial myopathies [progressive external opthalmoplegia (PEO) and mitochondrial encephalomyopathy, lactic acidosis and stroke-like episodes (MELAS)], acute quadriplegic myopathy (AQM) and chronic fatigue syndrome (CFS) all exhibit impaired metabolism, preferential loss of thick filaments and altered excitability of muscle. While the mitochondrial myopathies-PEO and MELAS are caused by mutations in mitochondrial DNA (MT-TL1 gene), AQM and CFS are idiopathic. The neuromuscular diseases-amyotrophic lateral sclerosis (ALS), spastic paraplegia (SP) and cerebral palsy (CP) all affect muscle secondary to neurodegeneration and are characterized by spasticity and progressive weakening of muscle.

**Table 1:** Diseases affecting muscle.

**Major disease category**	**Disease**	**Extant evidence for genetic association**	**GEO series**
*Muscular Dystrophy*	Becker muscular dystrophy (BMD, Dystrophinopathies)	DMD	GSE3307
	Duchenne muscular dystrophy (DMD, Dystrophinopathies)	DMD	GSE3307, GSE6011
	Emery Dreifuss muscular dystrophy (EDMD)	STA (EDMD1), LMNA (EDMD2)	GSE3307
	Facioscapulohumeral muscular dystrophy (FSHD)	FSHMD1A (rearrangement in subtelomeric region of 4q35)	GSE9397, GSE10760
	Limb-Girdle muscular dystrophies (LGMD) Type 2A	CAPN3	GSE3307, GSE11681
	LGMD Type 2B	DYSF	GSE3307
	LGMD Type 2I	FKRP	GSE3307
*Inflammatory Myopathies*	Polymyositis (PM)	Mostly idiopathic with evidence for association with HLA alleles	GSE3112
	Dermatomyositis (DM)	Mostly idiopathic with evidence for association with HLA alleles	GSE5370
	Juvenile dermatomyositis (JDM)	Mostly idiopathic with evidence for association with HLA alleles	GSE3307, GSE11971
*Inclusion body Myopathies*	Inclusion body myositis (IBM)	Mostly idiopathic with evidence for association with HLA alleles	GSE3112
	Hereditary inclusion body myopathy (HIBM)	GNE, MYH2	GSE12648
*Metabolic disorders affecting muscle*	Mitochondrial encephalopathy, lactic acidosis, and stroke-like episodes (MELAS)	MT-TL1	GSE1462
	Acute quadriplegic myopathy (AQM, Endocrine myopathies)	Idiopathic	GSE1017
	Chronic fatigues syndrome (CFS)	Idiopathic	GSE14577
	Progressive external opthalmoplegia (PEO)	MT-TL1 and/or POLG, SLC25A4, and C10orf2	GSE1017
*Neural diseases affecting muscle*	Amyotrophic lateral sclerosis (ALS)	C9orf72, SOD1, TARDBP, FUS, ANG, ALS2, SETX, VAPB (familial); idiopathic (sporadic)	GSE3307
	Hereditary spastic paraplegia (SP)	ATL1, SPG4, SPG20, SPG7	GSE3307
	Cerebral Palsy (CP)	Mostly idiopathic	GSE11686
*Other*	Sarcopenia		GSE1428

*This table represents the 20 muscle diseases considered in the current study along with the major disease category, current evidence for genetic association and the Gene Expression Omnibus (GEO) accession of the studies corresponding to muscle diseases. Sarcopenia was not included in any major disease category as it is an age related loss of muscle tissue*.

Despite observable physiological similarities in diseases within each group, diseases exhibit considerable heterogeneity in intensity, etiology, phenotypic manifestation, and gene expression. For instance, muscle in both inclusion body myopathies (HIBM and IBM, Table 1) exhibit chronic inflammation with visible vacuoles, however, IBM is mostly idiopathic, while mutations in GNE and MYH2 cause HIBM (Tomé and Fardeau, 1998). Systems biology, in particular network theory, facilitates an understanding of the heterogeneity and uniqueness underlying diseases mechanisms by integrating multiscale data (transcript, protein, and drug). Here we utilized a co-expression based scoring scheme to generate a network and elucidate mechanisms underlying significant disease-relevant modules. We further mapped them onto a “functional module” framework within muscle and onto a human protein interaction network, to infer a common program of regulation underlying majority of the diseases. Further, incorporating drug data into the quantitative framework allowed for identifying avenues for drug repurposing in treating diseases of the muscle.

## Materials and methods

### Data acquisition and processing

The list of diseases available under the Medical Subject Headings (MeSH) terms “neuromuscular,” “musculoskeletal,” and “muscular” diseases was used as a guideline for identifying muscular diseases of interest (Lipscomb, 2000). All available (RNAseq + microarray platform) information from GEO (Barrett et al., 2013) was downloaded and surveyed for maximum coverage of muscle diseases in the MeSH headings identified above. A single platform GPL96 (Affymetrix HG-U133A) offered the highest coverage of muscle diseases surveyed. Choosing studies from one platform alone (GPL96) limited possible noise arising from platform differences. Additionally, studies with non-muscle sample tissue, and less than two-samples/condition were eliminated. Filtering the data for accuracy, and experimental context using our constraints resulted in microarrays from 19 human diseases. In addition to these 19 diseases, samples from “CP” (not available under the MeSH term categories considered above) was also included resulting in 20 diseases for analysis. CP is a movement disorder characterized by contractures of the muscle with its primary insult on the nervous system. Gene expression data for studies with. CEL files (disease and control) are normalized using RMA (Robust Multi-array Average). Studies with series matrix files were downloaded as is. ComBat cross-array normalization is utilized for diseases with more than one associated GSE (e.g., LGMD2A, DMD, and JDM, Table 1), to remove study artifacts (Johnson et al., 2007). Multiple probes were accounted for using the “collapseRows” function of WGCNA library in R (Langfelder and Horvath, 2008). The reduced and processed data sets subsequently included z-transformed expression values of 12,789 genes across 20 diseases.

R (v 3.2.2) (R Core Team, 2015)/Bioconductor (Gentleman et al., 2004) was used for all data processing and pipelines implemented in this analysis.

### Identifying disease similarity

Cyber-T's (Kayala and Baldi, 2012) regularized *t*-test performed on z-transformed expression values was used as a measure of a gene's differential activity. The associated T-statistic provided insight into the difference in mean expression of a gene across conditions and was referred to as the differential gene activity (DGA) score for each disease state (Table S0). Partial pearson correlation of DGA scores across diseases, quantified disease similarity based on expression profiles. Use of partial correlation have been shown to be effective in factoring out dependencies such as variation in tissue types and experimental conditions (Suthram et al., 2010). The “pcor” function available through the “ppcor” package in R (Kim, 2015) was utilized to calculate all possible pairwise partial correlations between each pair of samples (here diseases) while eliminating the effect of all other samples. We identified disease clusters using hierarchical (complete-linkage) clustering of partial correlation.

### Disease-gene based disease overlap

A comprehensive list of genetic factors affecting the 20 muscle diseases from various sources such as OMIM (Amberger et al., 2009), PheGenI (Ramos et al., 2014), ClinVar (Landrum et al., 2014), and DisGeNET (Piñero et al., 2015) (henceforth referred to as the disease-gene list) was downloaded and curated. A hypergeometric model, with a null that disease-genes were randomly drawn from the space of all genes was used to ascertain the statistical overlap between diseases based on the disease-gene list. The function “phyper,” available through base R packages was utilized to calculate the hypergeometric *p*-values.

### Muscle “functional modules” and functional module activity score

We developed a framework of “functional modules” (FM) within muscle that represented significant units required for normal muscle activity (Table 2). Each of the 23 FMs were represented by a group of manually curated list of biomarkers that belonged to a broader functional pathway (family) within muscle (Table S1). This list expanded on an existing framework for muscle functional families (Mukund et al., 2014). Several biomarkers within each FM were multi-functional and were placed in FMs that were most relevant to skeletal muscle. The functional module activity (FMA) score associated with each functional module *i* in disease *k*, was calculated as mean DGA score of its component genes. This score reflected the overall state of a module in a particular disease (Table S2). Specifically, a negative FMA values reflected a downregulation of the module genes in disease with respect to controls and the converse for positive FMA values. Significance testing at *p* < 0.05 identified FMs associated with each disease state.

**Table 2:** Functional modules in muscle.

**Family**	**Functional module**	**ID**
*Neuromuscular Junction (NMJ)*	Components of the NMJ	1
	Synaptic basal lamina	2
*Excitation Contraction coupling (ECC)*	Ion Channels of post synaptic muscle	3
	Ion transporters pumps/exchangers	4
	Calcium dynamics/homeostasis required for ECC	5
*Contraction*	Sarcomeric thin filament associated	6
	Sarcomeric thick filament associated	7
	Sarcomeric z-disc associated	8
*Cytoskeleton*	Cytoskeleton	9
*Extracellular Matrix (ECM)*	Components of ECM	10
*Mitochondrial energy metabolism*	Glycolytic metabolism	11
	Oxidative metabolism	12
	Mitochondrial electron transporters	13
	Small molecule transporters	14
	Members of outer and inner mitochondrial membrane	15
	Associated signaling	16
*Hypertrophy*	Hypertrophy	17
*Atrophy*	Atrophy	18
*Inflammation*	Inflammation	19
*Regulators*	Myogenic and cell cycle regulators	20
*Fiber type maintenance*	Fiber type maintenance	21
*Vasculogenesis*	Angiogenic processes	22
*Oxidative stress*	Oxidative stress	23

*This table represents the 23 functional modules identified as belonging to 13 main functional pathways (families) associated with muscle*.

### Human protein interaction network and protein module activity score

The human protein-protein interaction network (PPIN) was extracted from the STRING database (v9.1), containing both direct (physical) and indirect (functionally derived) interactions (Franceschini et al., 2013). Limiting the interactions to a combined score cutoff of >0.85 allowed us to account for strong interactions, with sufficient experimental evidence and resulted in a total of 1,48,030 unique interactions among 10,341 proteins. The PPIN was clustered using MCL (Markov Cluster) algorithm a fast, scalable and unsupervised cluster algorithm for networks based on simulation of stochastic flow. The MCL algorithm finds cluster structure in graphs by iteratively computing the probabilities of random walks through the graph (markov matrices) using an alternation of two operators called expansion and inflation. Expansion is the power of a stochastic matrix using the normal matrix product (i.e., matrix squaring) and inflation is the Hadamard power of a matrix (taking powers entrywise), followed by a scaling step. A detailed description and comparison are provided in Enright et al. (2002) and Brohee and Van Helden (2006). Clustering the PPIN using MCL clustering resulted in 1,025 protein modules (filtered for minimum module size >2) with sizes ranging between 3 and 256 genes/proteins. We considered 764/1,025 modules (6,215/8,581 proteins overlapping with our list of 12,789 genes) with at least three genes/module for further analysis (Table S3). Analogous to Suthram et al. (2010) the protein module activity (PMA) score was calculated for each protein module *i* in a disease *k* as the mean of DGA scores for its component genes. In the end, we obtained a vector of PMA_ik_ for each disease, representing the activity level of a given protein module in each disease state (Table S4). Significance testing identified protein modules with a *p* < 0.05 associated with each disease. We defined the threshold for module expression as upper 50th percentile of |mean PMA| scores from significant modules across diseases.

### Significance testing

A background distribution of disease correlations expected at random was generated to assess the significance of observed correlations. Disease and control sample labels were shuffled prior to computing DGA scores and disease similarity (through partial correlations). The whole process was repeated 100 times to create a background distribution of disease correlations. This was utilized to determine a permutation-based *p*-value (number of the times the permuted statistic exceeded the observed statistic). The background distributions for PMA and FMA scores were similarly generated.

### Network visualization and functional enrichment

All network visualization was performed using Cytoscape software (Shannon et al., 2003). Enrichment was identified using Gene Ontology's -Biological Process category available via ClueGO-a Cystoscape plugin (Bindea et al., 2009) and DAVID v6.8 (Dennis et al., 2003). Venn diagram was created using the “VennDiagram” package available through R/Bioconductor (Chen and Boutros, 2011).

### Drug data

We utilized the drug gene interaction database (DGIdb) (Wagner et al., 2015) to identify a list of expert-curated proteins/genes that serve as druggable targets. A list of currently approved drugs from the FDA (or at least one jurisdiction) was downloaded from Drugbank (Law et al., 2014), while drugs treating the disease clusters were obtained from Medscape (Medscape, 2017)^1^ and UpToDate® (UpToDate, 2017)^2^

## Results

### Clustering muscle diseases

**Figure 1:**
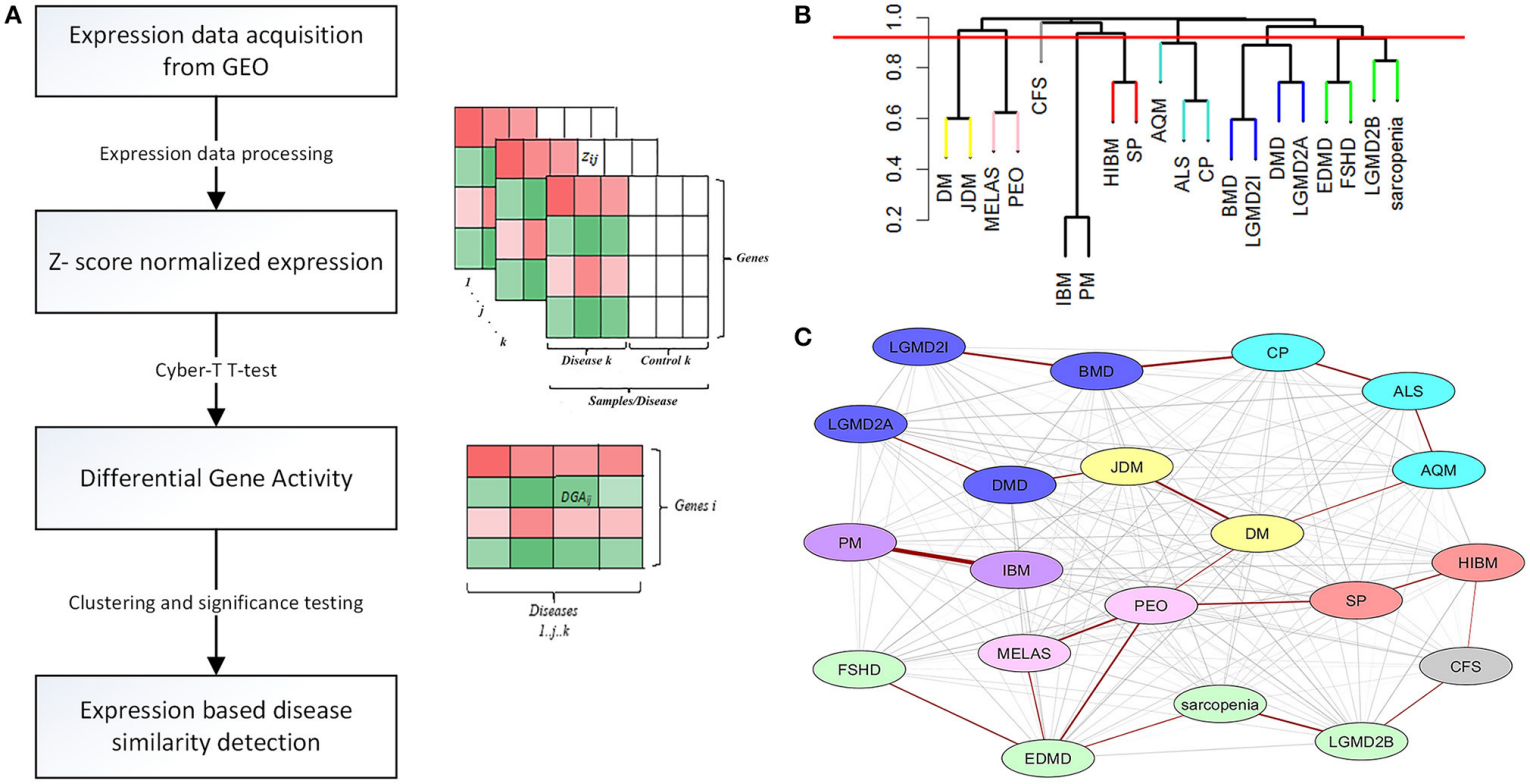
Extracting significant disease similarities from 20 diseases affecting muscle. **(A)** shows the workflow involved in calculating the differential gene activity (DGA) score and hierarchical clustering of the scores to extract disease clusters based on DGA. **(B)** shows the hierarchical clustering dendrogram (method- complete) of disease correlations. Tree cut height (red line) corresponds to a *p*-value of 0.05 and disease clusters identified below this line were identified to be significantly correlated. **(C)** This network represent the 190 possible associations between the 20 diseases. Edges highlighted in red indicate the associations identified as being highly significant through permutation testing. The various node colors indicate the clusters the diseases belong to as identified through hierarchical clustering. The nodes colored in gray were not clustered.

Expression data for diseases affecting muscle (Table 1) was processed (see Methods) resulting in a set of 12,789 genes across the 20 diseases and utilized in our analysis. The change in gene expression for each disease state (with respect to controls) was quantified as the associated T-statistic of z-normalized values (see Methods, Figure 1A, Figure S1) and referred to as differential gene activity (DGA) score. Hierarchical clustering of diseases based on partial correlation of DGA scores resulted in seven disease clusters (Figure 1B, Table 1). Twenty (~10%) of the 190 possible disease pairs were identified as being significant (*p* < 0.05) via significance testing (see Methods, Figure 1C). Few well-characterized intra-cluster associations marginally missed the significance threshold and were not captured, for instance the association between LGMD2B and DMD, BMD and DMD. We ascertained if the observed significant disease correlations (based on DGA scores) shared known genetic associations. We compiled a list of genes associated with our disease set from publicly available databases (see Methods). Pairs of disease were considered to significantly share disease-genes if the hypergeometric *p*-value of overlap was < 0.05. We identified that 26/190 possible disease pairs shared a significant genetic basis, and 6/26 interactions overlapped with our 20 significant DGA based associations (Table 3A), one-sided Fisher's exact test *p*-value of 0.036 (Table 3B).

**Table 3:** Disease association overlap.

**A**.					
**Disease 1**	**Disease 2**	**Hypergeometric *p*-value**		
DM	JDM	3.54E-12		
DMD	JDM	2.08E-02		
DMD	LGMD2A	9.09E-03		
BMD	LGMD2I	5.38E-03		
MELAS	PEO	6.95E-04		
IBM	PM	7.26E-10		
**B**.				
		**Correlations based on DGA scores**		
		**Significant**	**Not significant**	**Total**
**Correlations based on known disease genes**	**Significant**	6	20	26
	**Not Significant**	14	150	164
	**Total**	20	170	190

*A. This table provides associations that overlap between associations calculated based on the DGA scores and associations that share a genetic basis (disease-gene list based). Hypergeometric p-values of the overlap are also presented. B. Contingency table to evaluate the hypothesis that significant disease associations also significantly shared disease genes*.

### Identifying protein modules underlying muscle diseases

Protein modules identified within a human-PPIN through modularity detection algorithms (Enright et al., 2002; Brohee and Van Helden, 2006) represented a group of strongly interacting proteins with putative functional associations. We utilized PPIN to examine if there was a common program of regulation underlying different pathologies.

**Figure 2:**
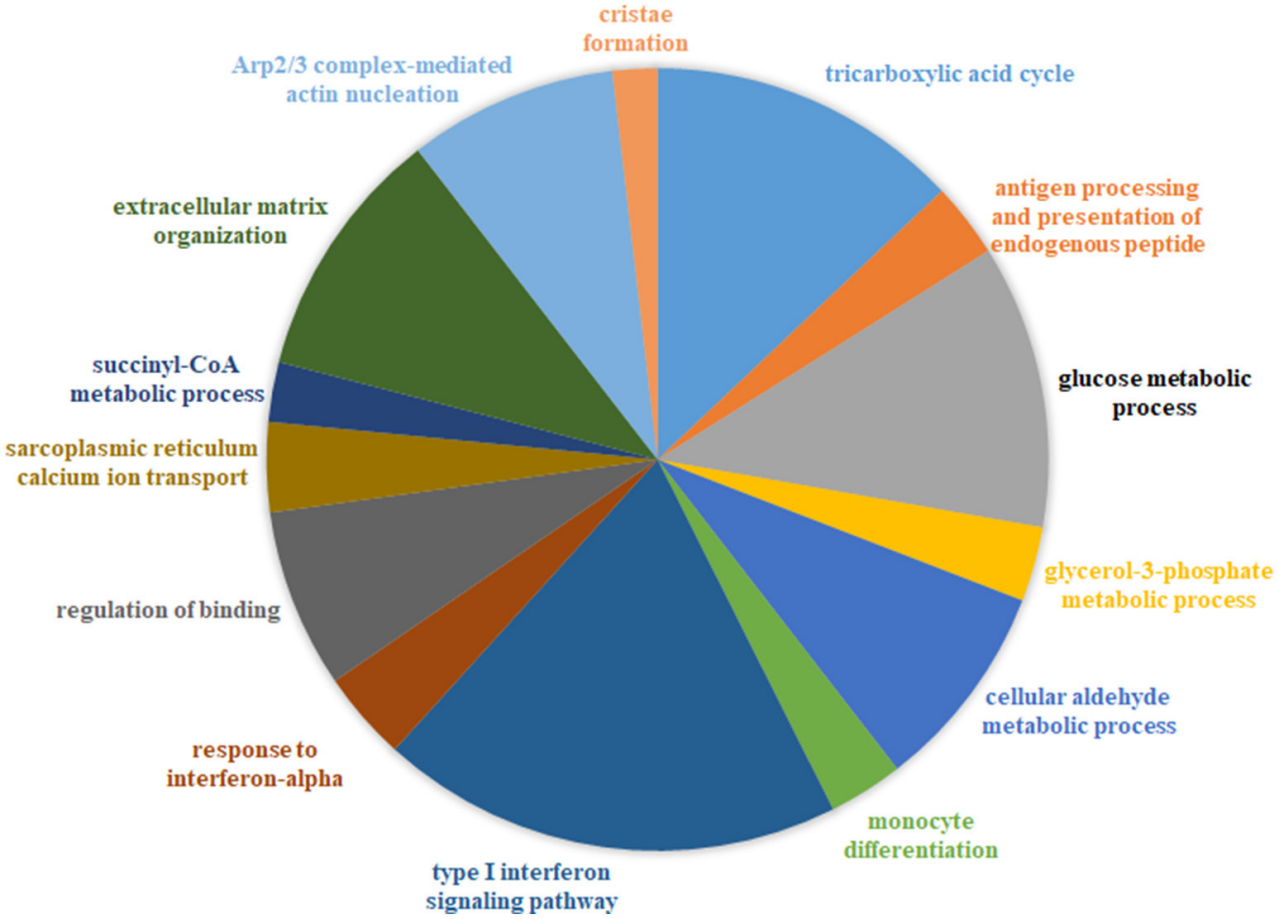
Combined functional enrichment of protein signature underlying diseases affecting the muscle. This figure provides a graphical representation of the top enrichment terms identified in the 17 signature protein modules (combined). The signature modules represent a set of modules that were identified as underlying a majority of the diseases considered in this study Size of each section of the pie is proportional to the number of genes identified in each category.

We generated a catalog of 1,025 protein modules by querying a large-scale human PPIN available through STRING (Franceschini et al., 2013) (see Methods) We identified that 764/1,025 modules (containing at least 3 nodes/module), overlapped our list of 12,789 genes, with module sizes varying from 3 to 256 proteins. We defined a protein module activity (PMA) score calculated for each protein module identified per disease as the mean of component protein DGA scores. The common underlying program of regulation or “signature” protein modules were identified utilizing a 2-fold approach-first, all modules with absolute PMA values significantly higher than random (*p* < 0.05) in more than half the diseases (*n* > 10) were extracted (35 modules passed this selection criterion). Next, using the threshold for module expression (see Methods), 17/35 modules were identified as the underlying protein signature (Figure 2, Table S5). The complete list of signature modules identified and their top three enrichment terms are provided in Table S6.

### A “functional module” framework to identify muscle-specific mechanistic changes

Utilizing the human PPIN allowed us to infer a common program of dysregulation underlying muscle diseases. We next sought to elucidate mechanistic similarities between significant disease pairs, in a more skeletal muscle-specific context. We developed a framework of FM that represented significant units required for normal muscle activity (Table 2, see Methods). The set of 23 “FM” captured key biomarkers associated with major processes in skeletal muscle. We calculated a functional module activity (FMA) score that reflected the collective behavior of genes in each functional module, for a given disease state (Table S2). We also computed an associated *p*-value for each functional module via permutation testing.

The FM framework was utilized to assess common functional mechanisms underlying 20 significant disease-pairs (with *p* < 0.05). Table 4 presents a subset of significant disease-pairs, which shared four or more significant FM between them. For instance, JDM and DMD had 15/23 FM overrepresented (*p* < 0.05). These included modules associated with atrophy, inflammation, ECM and cytoskeleton, all members of the excitation contraction coupling family (FM IDs-3,4,5), members of contraction (7,8), mitochondrial energy metabolism (11,12,13,14,15), inflammation, and fiber type maintenance (19 and 21).

**Table 4:** A representative set of functional modules shared between significant disease pairs.

**Significant disease association**	**Overlapping functional modules**
*ALS-CP*	2,5,12,13,14,15
*DMD-BMD*	5,7,10,12
*DM-JDM*	5,6,7,8,9,11,12,13,14,15,19,21
*EDMD-FSHD*	5,6,11,13
*IBM-PM*	5,9,10,11,12,15,16,21,23
*JDM-DMD*	3,4,5,7,8,9,10,11,12,13,14,15,18,19,21

*This table represents the functional modules identified as overlapping (p < 0.05) between the significant diseases associations identified on the left. The overlapping functional modules are identified using the IDs presented in Table 2*.

### Drug targets over-representation in disease-associated protein modules

**Figure 3:**
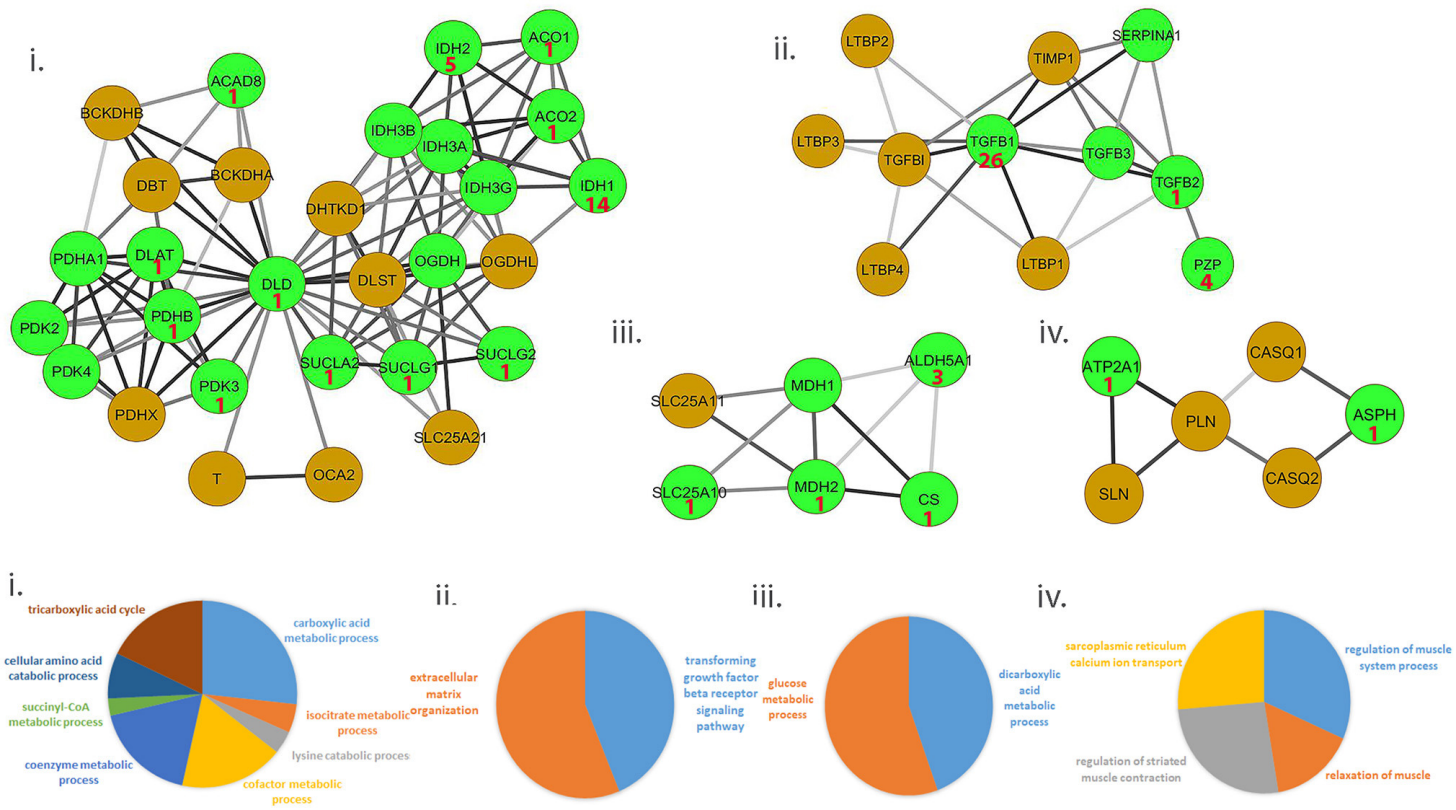
Representative set of the protein signature modules underlying diseases affecting the muscle. Top panel (i–iv) represents 4/17 protein signature modules identified. The green nodes represent proteins that contain at least one interaction as defined in the Drug-gene interaction database (DGIdb). Number shown in the nodes represent the number of approved drugs targeting the proteins. Lower panel (i–iv) represents functional enrichment (top group terms) identified for the corresponding modules panel (i–iv) in the top panel, using ClueGO (see Methods). All 17 modules identified are presented in Figure S2.

We ascertained if druggable targets were over-represented in the common protein signature modules to support the hypothesis that drugs targeting common targets can treat a variety of muscle diseases. We identified 54/156 proteins in the signature modules belonged to at least one druggable category, with at least one interaction as categorized in the drug gene interaction database (Wagner et al., 2015). We identified 41 of these proteins as targets for 81 approved drugs used in treating a variety of diseases and provided possible avenues for exploration of therapeutic options (Figure 3, top panel; Table S7).

We next hypothesized that protein modules exclusively regulated in each disease cluster might be enriched for drug targets. For instance, we identified protein modules associated with 3 disease clusters (DM/JDM, IBM/PM and DMD/BMD/LGMD) using an approach similar to section Identifying Protein Modules Underlying Muscle Diseases. We identified −20 approved drugs to be targeting proteins regulated in modules unique to the IBM/PM cluster, 26 drugs targeting proteins unique to the DMD/BMD/LGMD cluster and 203 in the DM/JDM cluster (Table S8).

## Discussion

### Clustering diseases based on differential gene activity identifies both well and less characterized disease associations

The seven disease clusters identified included several well-characterized clusters such as muscular dystrophies (BMD, DMD, LGMD2I, and LGMD2A), mitochondrial disorders (MELAS and PEO); and a few less characterized clusters such as Sarcopenia, EDMD, LGMD2B and FSHD. Fisher's exact test was performed to capture the significance of overlap between disease pairs identified using DGA scores and those with shared genetic basis (using disease-gene lists). The Fisher's exact *p*-value of 0.036 implied that the genetic disease similarity was significantly captured by DGA based correlations in this study. A relatively low, albeit, significant *p*-value of fisher's exact test reflected the possibility that certain disease associations did not pass our significance threshold or were not captured using disease-gene lists. This could also be attributed to certain diseases being better studied than others. For example, genes associated with ALS (895 genes) where far greater than those associated with AQM (26 genes).

### Deficient bioenergetics and calcium homeostasis–a common program of dysregulation underlying diseases affecting the muscle

Enrichment analysis of 17 signature modules, identified from the protein interaction network indicated an over-representation of modules associated with immune response (e.g., module 14), mitochondrial function (e.g., modules 41, 168, 271, 355, and 340), mitochondrial structure (e.g., 537), metabolism (e.g., 426), calcium homeostasis in muscle (e.g., 334), and the extracellular matrix (e.g., 153, 416) (Figure 2, Table S3).

Early mitochondrial research in muscle disorders have suggested that a widespread occurrence of mitochondrial anomalies did not necessarily imply a primary deficiency in efficacy of mitochondrial function (muscle meeting its energy requirements) (Stadhouders and Sengers, 1987). However, more recent research has repeatedly suggested deficient bioenergetics underlie the pathology of several muscular and neuromuscular diseases in mammalian models (Wallace, 2000, 2013; Pieczenik and Neustadt, 2007; Ramadasan-Nair et al., 2014). Pathology of neuromuscular diseases such as ALS also exhibits mitochondrial dysfunction as a major event in its progression (Dupuis and Loeffler, 2009; Cozzolino and Carrì, 2012). Reduced efficiency in the action of the tricarboxylic acid (TCA) cycle has been also assessed in diseased muscle associated with inflammatory myopathies (Coley et al., 2012), dystrophy (Even et al., 1994), and mitochondrial diseases such as MELAS, PEO (Wallace, 1999). Mounting evidence has suggested that the pathological muscle wasting observed in dystrophies (e.g., DMD) might be due to reduced ATP availability required for maintenance of Ca^2+^ homeostasis and fiber regeneration (Timpani et al., 2015). Bioenergetic pathway enzymes have recently shown to be relevant biomarkers of muscular and neuromuscular disease progression (Santacatterina et al., 2015).

Ca^2+^ homeostasis in muscle largely determines its contraction and relaxation properties. This is tightly regulated by the Ca^2+^ signaling apparatus within muscle comprising of the ryanodine receptors, sarcoplasmic endoplasmic reticulum calcium pumps (SERCA), troponin complex, calsequestrin; in addition to Ca^2+^ binding proteins such as parvalbumin, sarcolipin, phospholamban and calpains. We observed a strong dysregulation of several of these proteins- ATP2A1 (SERCA pump), sarcolipin (SLN, which inhibits SERCA) and calsequestrin [CASQ, restrains Ca^2+^ to the sarcoplasmic reticulum (SR)], in ALS and DMD (Wang et al., 2012; Mukund and Subramaniam, 2015). Likewise, regulation of ASPH (regulator of ryanodine receptors) and SLN have also been observed in muscle from diseases such as CP (Smith et al., 2011). Figure 3 provides a representative sample of 4/23 signature modules and their enrichment.

Although existing research on several muscle diseases (such as ALS, DMD, BMD, and CP) has shown varying extents of mitochondrial dysfunction and calcium dysregulation in their pathomechanism, our approach points to widespread, statistically non-random dysregulation of mitochondrial function and calcium homeostasis associated with most muscle diseases including relatively less characterized diseases such as AQM and CFS. Further, the absence of modules associated with structural sarcomeric proteins (myosins, z–disc proteins, dystroglycan) at our significance threshold emphasizes the vital role of muscle bioenergetics, calcium signaling and homeostasis pathways in the pathogenesis of diseases affecting muscle.

### Muscle specific mechanistic changes underlie disease pairs

The functional module framework utilized, allowed us to capture common functional mechanisms underlying disease pairs. Table 4 represented a subset of significant disease-pairs, which shared four or more significant FM between them (e.g., JDM and DMD having 15/23 FM overrepresented; *p* < 0.05). DMD and JDM represent myopathies, where the primary insult is on the skeletal muscle however, JDM is a systemic autoimmune vasculopathy characterized by weakness of proximal muscles and skin rashes with its histopathology showing evidence for necrosis, fiber size variation, and a muscle degeneration/regeneration phenotype (Peloro et al., 2001). JDM shares many pathologic similarities with muscle of children affected by DMD. A comparison of the expression profiles of children with DMD and JDM have revealed similarities in gene cascades involving muscular atrophy, deficits in mitochondrial metabolism and contraction, along with upregulation of extracellular matrix and cytoskeletal cascades (Tezak et al., 2002) consistent with the functional overlap observed in our study (see Results).

Finding relevant FM consistent with the current understanding of similarities between JDM and DMD further justified the efficacy of the adopted approach in identifying FM affected in more than one diseases state, in a context specific manner. To further elucidate disease associations much less explored, we focused on two diseases ALS and CP and their overlapping FM and associated FMA scores.

#### Calcium dysregulation in patients with ALS and CP

ALS and CP, both represent neurological diseases with their primary insult on upper and/or lower motor neurons. While ALS is a neurologically progressive disease, CP is not, with both disorders exhibiting *progressive musculoskeletal* weakness and increased spasticity. While ALS muscle is additionally characterized by denervation atrophy and spasticity, there is distinctive shortening and subsequent weakness of CP muscle (Graham and Selber, 2003; Kiernan et al., 2011). We identified 6 FM as being significantly dysregulated in both ALS and CP (Table 4). The associated FMA scores reflected the state of FM in disease, specifically a negative FMA scores reflected a general downregulation of genes associated with the functional module in the particular disease.

We observed that 5/6 FM identified above were similarly regulated in both ALS and CP (Table 5), mainly associated with mitochondrial metabolism (FM IDs 12–15). There is abundant evidence in literature for mitochondrial dysfunction particularly electron transport chain dysregulation and its role in ALS (Borthwick et al., 1999; Crugnola et al., 2010) in neurons. Our results indicated similar programs of mitochondrial dysregulation to be associated with *muscle* in patients with ALS and CP. Though no detailed studies in muscle exist to corroborate mitochondrial dysfunction in CP, Smith et al. (Smith et al., 2009, 2011) also show a general downregulation of mitochondrial transcripts. Comparison of the expression values for genes associated with these FM showed an R^2^ of 0.9.

**Table 5:** Overlapping functional modules between ALS and CP.

**ID**	**Functional module**	**ALS FMA score**	**CP FMA score**
2	Synaptic basal lamina	1.42	1.53
5	Calcium dynamics/homeostasis required for ECC	−2.02	1.72
12	Oxidative metabolism	−2.62	−2.09
13	Mitochondrial electron transporters	−3.17	−2.14
14	Small molecule transporters	−2.99	−1.94
15	Members of outer and inner mitochondrial membrane	−1.18	−1.34

*This table provides a list of functional modules that were identified as being significantly shared between two diseases ALS and CP along with their computed functional activity scores*.

Cellular dysregulation of Ca^2+^ dysregulation in ALS within affected *neurons* is well-characterized (Grosskreutz et al., 2010); likewise, dramatic Ca^2+^ dysregulation within *muscle* from CP patients has also been suggested to occur (Smith et al., 2009, 2011). Interestingly, the FMA scores indicated a differential regulation of calcium dynamics/homeostasis in *muscle* from ALS and CP (Table 5). A few notable differences identified in the differential regulation of ALS and CP FM were as follows- ATP2A1 and ATP2A2, two-muscle specific, energy demanding fast fiber SERCA pumps (sarco (endo) plasmic reticulum Ca^2+^ ATPase) were very strongly downregulated in ALS, suggesting a reduced efficacy in sequestering Ca^2+^ to the SR from the cytosol (Periasamy and Kalyanasundaram, 2007). The reduced need for regulation of SERCA pumps was reflected in the downregulation of its two strong regulators- SLN and PLN. Upregulation of ryanodine receptors (RYR3) further emphasized leakage of SR Ca^2+^ into the cytosol (Perez et al., 2005). Activation of non-skeletal muscle isoforms pointed to a shift in fiber composition toward a slower/more mixed phenotype in ALS.

**Figure 4:**
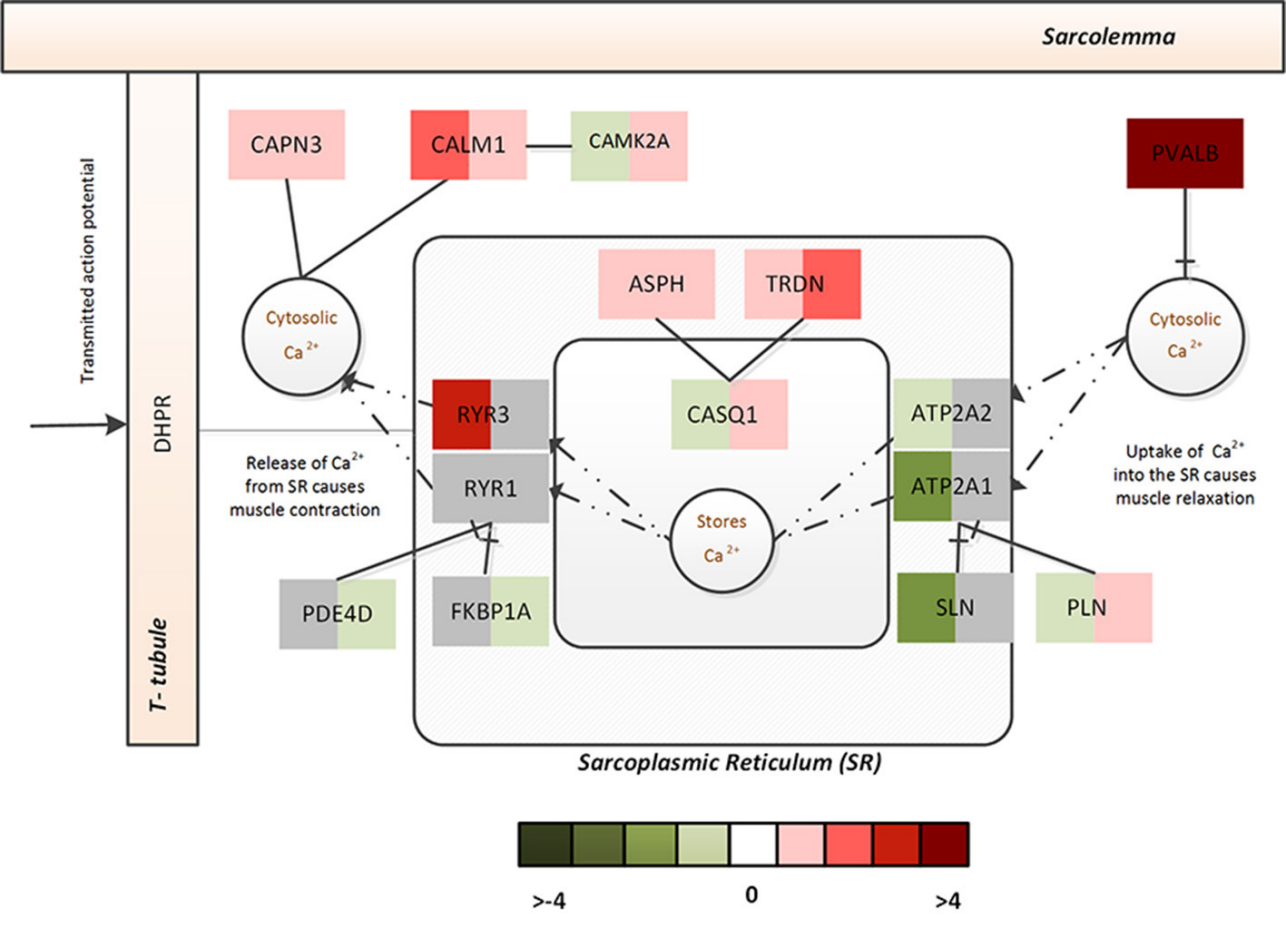
The Ca^2+^ homeostasis associated functional module in ALS and CP. This figure captures the calcium dysregulation mechanisms (and difference between ALS and CP) via the fold changes associated with select genes of the Ca^2+^ homeostasis functional module. The left half indicates the fold change associated with ALS while the right half indicates the fold change associated with CP.

In CP, increased Ca^2+^ was inferred from the massive upregulation of PVALB, which selectively binds to free Ca^2+^ to reduce free intracellular Ca^2+^ (subsequently, bringing about muscle relaxation). Though no significant changes were observed with respect the SERCA pumps or ryanodine receptors, FKBP1A and PDE4D that prevent channel leaking were significantly downregulated and PLN that controls the Ca^2+^ intake by the SERCA pumps was significantly upregulated in CP. On the other hand, upregulation of ASPH, TRDN, and CASQ1 indicated that muscle was actively trying to sequester intracellular Ca^2+^ to the stores. Figure 4 represents associated fold changes for select genes from the calcium homeostasis functional module.

Taken together this indicates increased cytosolic Ca^2+^ in both diseases, however, in ALS- the Ca^2+^ homeostasis machinery associated with SR appears to be severely challenged by the disease with increased leakage of Ca^2+^ from the intracellular stores and a constrained uptake of Ca^2+^ back into SR. In contrast, CP displays a use-dependent decrease in capacity of the SR albeit muscle's efforts to actively recover its Ca^2+^ stores. This dramatic adaptation in both ALS and CP muscle might additionally lead to altered muscle contractile properties and mitochondrial functions.

### Exploring opportunities for drug-repositioning

Current advancements in understanding muscular/ neuromuscular disease pathophysiology have allowed for drastic improvements in drug therapy, however, several of the diseases discussed here are as yet untreatable with high rates of morbidity and mortality with limited therapeutic options. Gene therapy and precision medicine are yet to be realized in their full potential for several of the diseases considered here (Nightingale et al., 2016; Dalakas, 2017). Given this shortage of drug/therapeutic availability for muscle diseases, we aimed to identify if drug-repurposing opportunities could be inferred from our quantitative framework across the 20 diseases. We identified 81 approved drugs to be targeting proteins contained within the signature modules (Table S7) warranting further analysis.

Further, several of the available treatments for muscle diseases currently only offer symptomatic relief, for instance, no specific therapeutic treatments exist for dystrophies such as DMD, BMD LGMD, or EDMD, with patients requiring aggressive supportive care. These patients are only often treated for associated conditions of the heart and lung. Likewise for neurological diseases which are multi-symptomatic such as ALS and CP, patients are mostly provided symptomatic relief with a multitude of agents such as antiparkinsonian, anticonvulsant, antidopaminergic, antispasticity, anti-sialorrhea, or antidepressants. This prompted us to further explore if there were drugs uniquely shared by disease clusters that could provide opportunities for drug repositioning within disease clusters.

**Figure 5:**
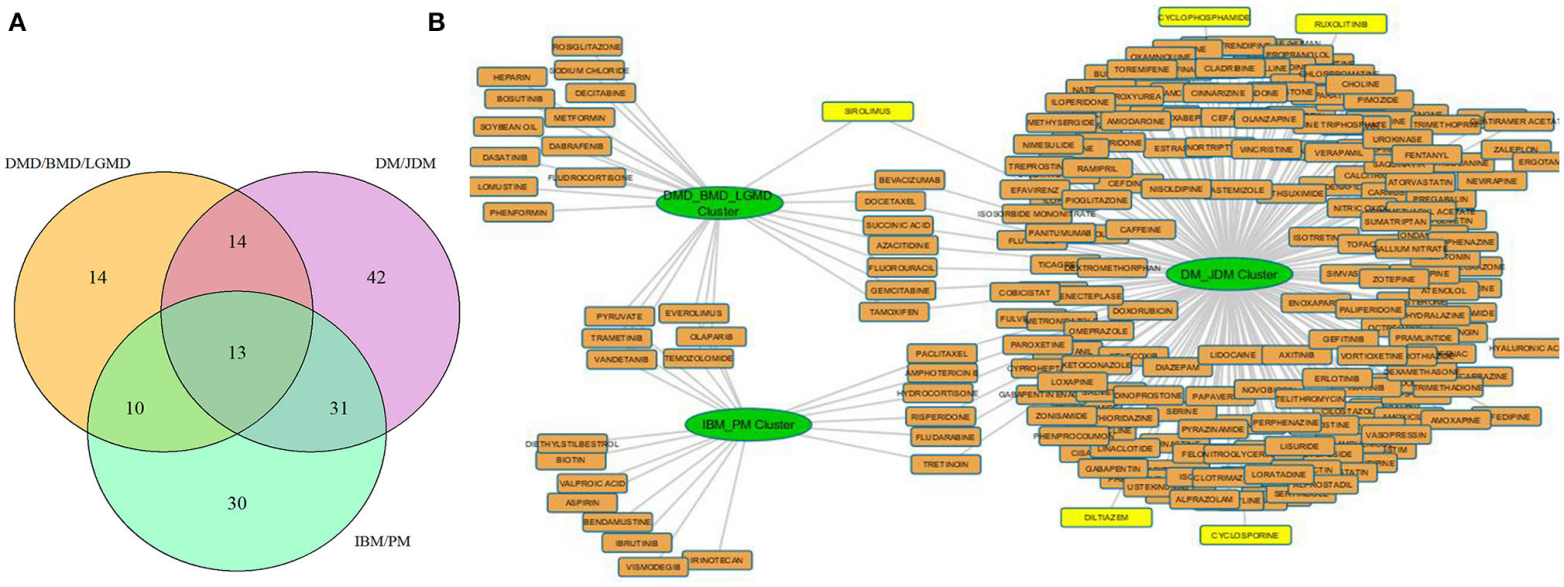
Drug disease network for 3 disease clusters. **(A)** shows the number of protein modules associated with each disease cluster considered e.g., 13 protein modules were shared among all clusters, 14 modules were uniquely regulated in the DMD/BMD/LGMD cluster, 30 in the IBM/PM cluster and 42 in the DM/JDM cluster. **(B)** represents the approved drugs (Table S8) associated with the protein modules uniquely regulated in each disease cluster. Nodes in yellow are drugs currently utilized for treatment in the diseases associated with the cluster. Sirolimus and Ruxolitinib, investigational therapeutics currently used in DM/JDM were also identified within the DM_JDM cluster.

Our results from three associated disease clusters (DM/JDM, IBM/PM and DMD/BMD/LGMD clusters) supported this hypothesis (Figure 5, Table S8), for example, several drugs such as diltiazem, cyclophosphamide, cyclosporine—prescribed for management of DM and JDM were identified associated within the DM/JDM cluster (“UpToDate” 2017)^3^. We also observed in the cluster presence of Ruxolitinib- a JAK inhibitor developed to treat neoplastic diseases but suggested as investigational therapy for DM (Hornung et al., 2014). Interestingly, Sirolimus a FDA approved drug for prophylaxis against organ rejection, is currently suggested for symptomatic relief in patients with DM (Nadiminti and Arbiser, 2005). Sirolimus appears to also target proteins associated in the DMD/BMD/LGMD clusters suggesting possible therapeutic opportunities in different muscle disease categories for Sirolimus (and possibly other shared drugs identified in Figure 5). These results further emphasized opportunities and a need for exploring repurposing of therapeutics in diseases affecting the muscle.

## Conclusions

Our study demonstrated the value of an integrated approach in revealing disease relationships and highlighted opportunities for therapeutic advancements in treating muscle diseases. Clustering of the co-expression network based on the differential gene activity-scoring scheme allowed us to identify disease clusters, not based on clinical or pathological similarity, but on the similarity of the expression profiles. A “FM” framework of 23 modules was developed to provide a muscle-context specific view of the mechanistic similarities. Integrating this with our data allowed for understanding less explored disease associations such as ALS and CP.

Incorporating protein information with the diseases similarity network allowed for identification of a “common signature”—a set of pathways underlying a majority of the diseases considered here. The common signature included pathways contributing to deficient bioenergetics and calcium dysregulation within affected muscle. An observed overrepresentation of druggable targets within these signature modules, in addition to subset of drugs uniquely associated with three disease clusters (DM/JDM cluster, IBM/PM cluster and DMD/BMD/LGMD cluster) further allowed us to recognize possible avenues for drug repurposing in treating diseases of the muscle.

## Author contributions

KM and SS conceived the study, reviewed and edited the manuscript. KM designed the study, performed the analysis and prepared the manuscript.

### Conflict of interest statement

The authors declare that the research was conducted in the absence of any commercial or financial relationships that could be construed as a potential conflict of interest.

## Footnotes

^1^https://reference.medscape.com/

^2^https://www.uptodate.com/contents/search

^3^https://www.uptodate.com/contents/search
