# Supplementary material for: Co-expression Network Approach Reveals Functional Similarities among Diseases Affecting Human Skeletal Muscle
Source: Front Physiol. 2017 Dec 1;8:980. doi: 10.3389/fphys.2017.00980 (PMC5717538; doi:10.3389/fphys.2017.00980)
Supplement: Supplementary file 2 [file Image1.pdf]

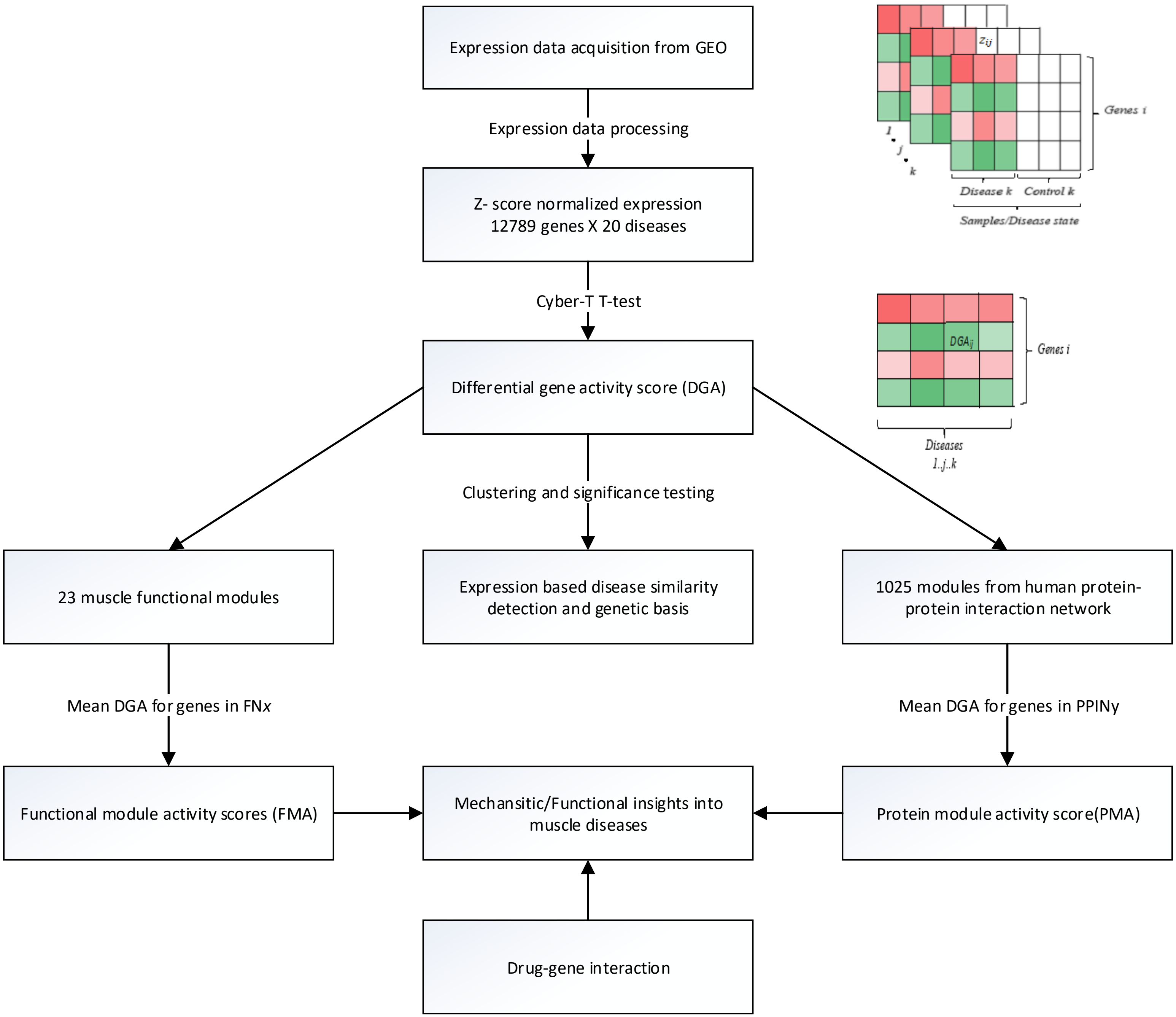

Figure S1: This flowchart represents the entire workflow adopted in our study.

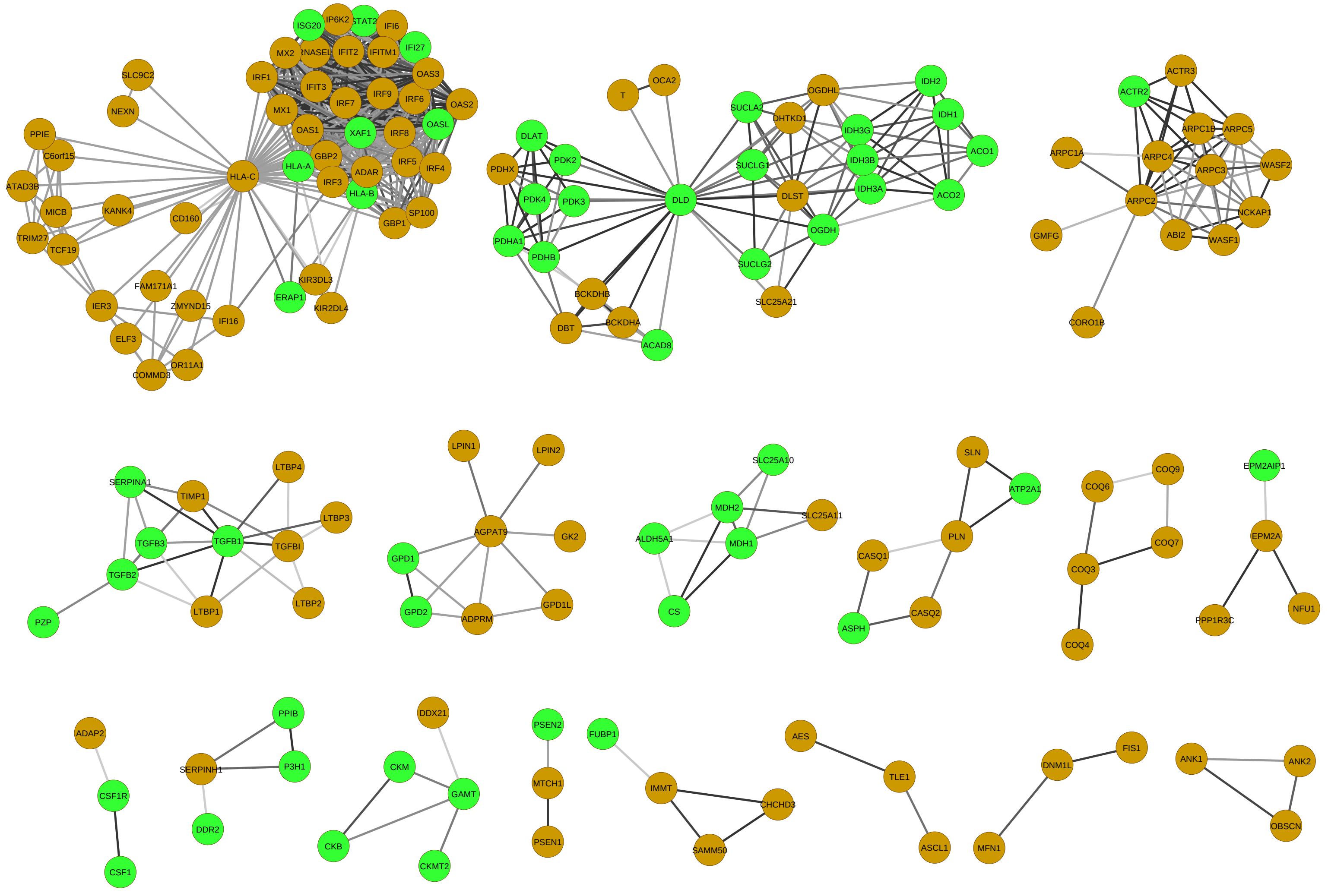

Figure S2- Represents all 17 modules identified as part of the "protein signature" underlying more than half the diseases considered in our study. Green nodes indicate genes that have atleast one drug interaction as obtained from the drug-gene interaction database DGidb.
